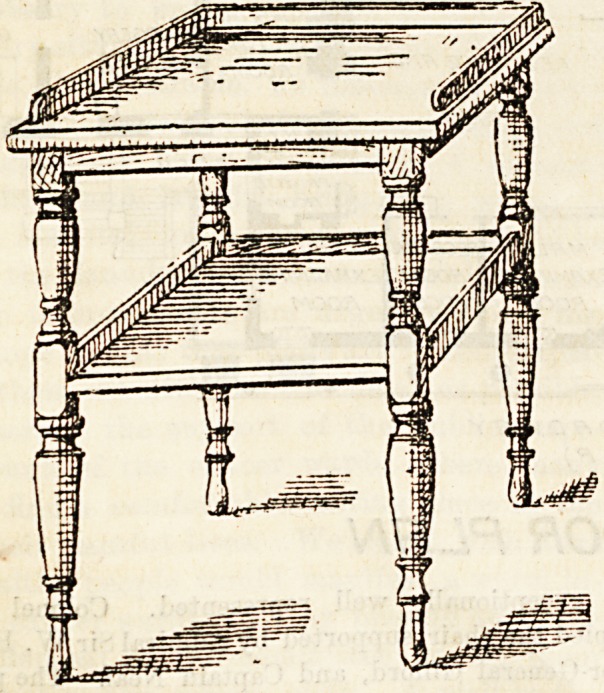# Glass-Topped Locker

**Published:** 1893-11-18

**Authors:** 


### PRACTICAL DEPARTMENTS.

### GLASS-TOPPED LOCKER.

The accompanying illustration of a small table or locker is given by kind permission of Messrs. Atkinson, of Westminster Bridge Road. Its special claim to notice lies in the use of a sheet of plate-glass for the top shelf or tray, which, for obvious reasons, is a cleanly and much to be recommended plan. For all ward tables and lockers tiles or glass should be used where practicable, as being kept easily cleaned and bright. An improvement, we may suggest, would be to provide castors, so that the locker may be moved with greater ease and convenience. We have before had occasion to mention the lockers made by Messrs. Atkinson, who exhibit many varieties, and have a special department in connection with ward furniture and fittings.

## Figures and Tables

**Figure f1:**